# Supplementary material for: Novel modelling strategies for high-frequency stock trading data
Source: Financ Innov. 2023 Jan 17;9(1):39. doi: 10.1186/s40854-022-00431-9 (PMC9842577; doi:10.1186/s40854-022-00431-9)
Supplement: Supplementary file 1 — Additional file 1. Table S1. Proportion of the outcome variable Y (Upwards/Stationary/Downwards) according to different α values and over three months trading time. Table S2. Proportion of the outcome variable Y (Upwards/Stationary/Downwards) according to different α values and over June 2017. Table S3. Proportion of the outcome variable Y (Upwards/Stationary/Downwards) according to different α values and over July 2017. Table S4 .Proportion of the outcome variable Y (Upwards/Stationary/Downwards) according to different α values and over August 2017. [file 40854_2022_431_MOESM1_ESM.pdf]

Supplementary Document - Novel Modelling Strategies for High-frequency Stock Trading Data

Xuekui Zhang[1*†], Yuying Huang[1,2†], Ke Xu[3] and Li Xing[4]

*Correspondence: xuekui@uvic.ca
[1]Math&Stat Department at University of Victoria, Victoria, Canada
Full list of author information is available at the end of the article
†Equal contributor

1 Introduction of Machine Learning Base Learners

1.1 Supporting Vector Machines

Supporting Vector Machines has gained its large popularity in the research area of financial data prediction, (e.g., [1], [2], and [3]). In this section, we will give a brief introduction to SVM model. For further details regarding model design and kernel choosing, (see [4], [5]).

We denote i-th input observation as $(x_i, y_i),\ y_i \in \{+1, -1\}$, where y is the binary response and x_i is the features vector of the individual observation. To construct the optimal hyperplane regardless of linear separability of the given data set, one first need to transform the p-dimensional input into a P-dimensional space using a projection $\phi : \mathbb{R}^p \to \mathbb{R}^P$. Our decision rules are as follows:

$$y_i(w^T\phi(x_i) + b) \geq 1 \tag{1}$$

where w is the normal vector of the hyperplane, and $b \in \mathbb{R}$ is the unknown parameter. The margin between the closest data points from both positive and negative sides sums as $\frac{2}{||w||}$, we can maximize the margin by minimizing $\frac{1}{2}||w||^2$. The optimal w and b are found using quadratic programming optimization to first find the α_i which maximize:

$$\sum_{i=1}^{N} \alpha_i - \frac{1}{2}\sum_{i,j}^{N} \alpha_i \alpha_j \phi(x_i)^T \phi(x_j)$$

where

$$\alpha_i \geq 0, \forall i, \sum_{i=1}^{N} \alpha_i y_i \geq 0 \tag{2}$$

We can solve the loss function when considering general forms of the dot-product in a Hilbert space, where a kernel function come to help to define the inner product of $\phi(x)$:

$$\kappa(x_i, x_j) = \langle \phi(x_i), \phi(x_j) \rangle \tag{3}$$

After we solve α_i, we get:

$$w = \sum_{i=1}^{N} \alpha_i y_i \phi(x_i) \tag{4}$$

and the set of support vectors $\mathbb{S}$ is represented by the indices i, where $\alpha_i > 0$. The bias b can then be calculated:

$$b = \frac{1}{N_s} \sum_{s \in \mathbb{S}} \left(y_s - \sum_{m \in S} \alpha_m y_m \kappa(x_m, x_s) \right) \tag{5}$$

For a new data x, the classi1cation function is then given by

$$f(x) = \text{Sign}(w^T\phi(x_i) + b) \tag{6}$$

In the implement of 3-category SVM model, SVM uses the 'one-against-one' approach: three binary classifiers are trained and the appropriate predictive class is found by a voting scheme.

1.2 Elastic Net Model

In a linear regression problem, a typical classic way of obtaining the vector of unknown coefficients is called the ordinary least squares (OLS), minimizing the residual sum of squares. Since OLS does not always perform well in many prediction and interpretation scenarios, penalization techniques have been proposed to improve OLS. Ridge regression model proposed by Hoerl and Kennard [6], introduced a size constraint, which is a L_2-norm of the coefficients, to the regularization, therefore alleviated the problem of the occurrence of high variance in the OLS estimated coefficients. However, ridge regression cannot produce a parsimonious model for it only shrinks the coefficients towards zero and each other when enlarging the amount of shrinkage but it does not eliminate any of the variables. Another promising shrinkage method called the Lasso was proposed by Tibshirani [7], which minimizes the residual sum of squares subject to a bound on the l_1 penalty. Lasso automatically selects the variables that contribute most to the model performance, shrinks the coefficients of those insignificant variables to zeroes and therefore yields a more explicable model. Lasso also has its disadvantage when it comes to multicollinearity, the Lasso tends to randomly select one variable from a group of highly correlated variables and ignores the others. In order to fix the problem about Lasso mentioned above and, Zou and Hastie [8] brought up a new regularization technique named the Elastic Net. The Elastic Net is an intermedia continuous shrinkage method between Ridge regression and Lasso regression since its regularization is a mixture of the l_1 penalty of Lasso regression and the l_2 penalty of Ridge regression. The introduction of both penalties have an impact on the estimation of the unknown coefficients. Hence, ENet likewise does automatic variables selection and simultaneously it can select group of correlated variables.

In the case where the response variable Y is three-classes categorical, ENet fits a multinomial logistic regression model, where the probability of each class of the i-th observation is represented by a linear function as follow:

$$Pr(Y_i = k) = \frac{e^{\beta_{k0}+x_i^T\beta_k}}{\sum\limits_{l\in\mathcal{D}} e^{\beta_{l0}+x_i^T\beta_l}}, \quad \text{for } k \in \mathcal{D} = \{\text{Upwards, Stationary, Dowardwards}\} \tag{7}$$

where x_i is the vector input of the i-th observation. β is a $p\times 3$ matrix of coefficients where $\beta_k = (\beta_{k1}, \dots, \beta_{kp})^T$ refers to the k-th column, which is a vector of p unknown coefficients for class k, and β_{k0} is the slope for class k. There is no closed-form solution to β in multinomial logistic regression model. As for implement of the ENet model, we use R function **glmnet** [9], to solve the complicated objective function, namely, the elastic net penalized negative log-likelihood function:

$$l(\{\beta_k\}_1^K) = -\left[\frac{1}{N}\sum_{i=1}^N \left(\sum_{j\in\mathcal{D}} y_{il}(\beta_{j0} + x_j^T\beta_j) - log(\sum_{l\in\mathcal{D}} e^{\beta_{l0}+x_i^T\beta_l})\right)\right] + \lambda\left[\frac{(1-\alpha^*)}{2}||\beta||_F^2 + \alpha^* \sum_{m=1}^p ||\beta_m||_1\right]$$

(8)

where $y_{il} = I(g_i = l)$ is the indicator function response, $||\cdot||_F$ is the Frobenius norm, λ is the complexity parameter that control the amount of shrinkage, and $\alpha^* \in [0, 1]$ is an ENet mixing ratio parameter that balances the quadratic and absolute penalty terms: when $\alpha^* = 1$, the ENet is actually LASSO regression and when $\alpha^* = 0$, the ENet turns into Ridge regression. The R function uses a so-called partial Newton algorithm to approximate the log-likelihood and therefore numerically derives β [9]. With the estimated coefficients, we can assess the most probable category given its observed features and make a new classification upon the input. In our experiment, we mainly consider the ENet with $\alpha^* \in [0.2, 0.8]$, given intricacy of correlation among our handcrafted features: variables of sequential prices are highly correlated while other variables are not. The details about choosing values of parameters α^* and λ in our ENet model are explained in the empirical application section.

2 Proportion of Y based of α

In this section, we summarize four tables about the proportion of the categorical outcome variable Y regarding different threshold values α. Table S1 is the summary over the whole trading period, Table S2, Table S3, and Table S4 are summaries over June, July, and August of 2017, respectively. In short, we find that when $\alpha = 10^{-4}$, all the stocks have an extreme data unbalance issue. This issue gets alleviated when α equals 10^{-5} or 10^{-6} for some stocks. The optimal threshold varies for different stocks. We would suggest that the users choose a proper threshold based on the stock's volatility and the distribution of the categorical responses.

Table S1 Proportion of the outcome variable Y (Upwards/Stationary/Downwards) according to different α values and over three months trading time.

	$\alpha = 10^{-6}$	$\alpha = 10^{-5}$	$\alpha = 10^{-4}$
AAPL	20/61/19	16/69/16	0/100/0
MSFT	7/86/7	7/86/7	0/99/0
MMM	37/27/37	28/45/28	1/98/1
AXP	14/72/14	14/72/14	1/98/1
BA	38/22/40	30/39/31	2/97/2
CAT	32/36/32	27/46/27	1/97/1
CVX	23/53/23	19/61/19	1/99/1
CSCO	5/91/5	5/91/5	2/96/2
KO	9/83/9	9/83/9	3/94/3
DOW	16/68/16	16/68/16	2/95/2
XOM	9/81/9	9/81/9	1/99/1
WBA	25/50/25	25/50/25	3/94/3
GS	43/13/44	34/31/34	3/95/2
HD	32/36/32	27/46/27	1/98/1
INTC	5/90/5	5/90/5	2/96/2
IBM	37/25/38	32/36/32	1/98/1
JNJ	25/50/25	21/59/21	1/99/1
JPM	9/82/9	9/82/9	0/99/0
MCD	34/32/34	29/43/29	1/98/1
MRK	11/79/11	11/79/11	2/97/2
NKE	10/80/10	10/80/10	2/97/2
PFE	5/89/5	5/89/5	3/95/3
PG	10/80/10	10/80/10	1/99/1
TRV	37/25/38	32/35/33	3/95/3
UNH	39/21/39	34/32/34	2/97/2
UTX	32/35/33	28/45/28	2/97/2
VZ	6/88/6	6/88/6	2/96/2
V	15/69/15	14/71/14	0/99/0
WMT	14/72/14	14/72/14	1/97/1
DIS	18/63/18	15/70/15	1/99/1

Table S2 Proportion of the outcome variable Y (Upwards/Stationary/Downwards) according to different α values and over June 2017.

	$\alpha = 10^{-6}$	$\alpha = 10^{-5}$	$\alpha = 10^{-4}$
AAPL	21/58/21	17/66/17	0/100/0
MSFT	8/85/8	8/85/8	1/99/1
MMM	35/31/34	25/50/25	1/99/1
AXP	12/76/12	12/76/12	1/98/1
BA	37/25/37	32/37/32	2/97/2
CAT	32/36/32	27/46/27	2/97/2
CVX	24/52/24	20/60/20	1/99/1
CSCO	4/91/4	4/91/4	2/96/2
KO	9/82/9	9/82/9	3/95/3
DOW	17/67/16	17/67/16	2/95/2
XOM	9/82/9	9/82/9	1/99/1
WBA	29/43/29	29/43/29	4/92/4
GS	43/14/44	34/32/34	3/94/3
HD	30/40/30	25/50/25	1/99/1
INTC	5/90/5	5/90/5	2/95/2
IBM	38/23/38	33/35/33	1/98/1
JNJ	20/59/20	17/67/17	0/100/0
JPM	9/82/9	9/82/9	1/99/1
MCD	33/34/33	28/44/28	1/98/1
MRK	11/78/11	11/78/11	2/97/2
NKE	9/82/9	9/82/9	2/97/1
PFE	6/88/6	6/88/6	3/94/3
PG	11/78/11	11/78/11	1/98/1
TRV	36/26/37	31/37/32	2/96/2
UNH	40/21/39	35/32/34	2/96/2
UTX	29/42/29	25/51/25	1/98/1
VZ	5/90/5	5/90/5	2/97/2
V	15/70/15	15/70/15	0/99/0
WMT	15/70/15	15/70/15	1/97/1
DIS	17/65/17	14/71/14	0/99/0

Table S3 Proportion of the outcome variable Y (Upwards/Stationary/Downwards) according to different α values and over July 2017.

	$\alpha = 10^{-6}$	$\alpha = 10^{-5}$	$\alpha = 10^{-4}$
AAPL	17/65/18	14/72/14	0/100/0
MSFT	6/87/6	6/87/6	0/99/0
MMM	37/25/38	29/42/29	2/97/2
AXP	15/69/15	15/69/15	1/98/1
BA	39/21/40	31/39/31	2/96/2
CAT	33/35/33	28/45/28	2/96/2
CVX	23/54/23	19/62/19	1/99/1
CSCO	5/91/5	5/91/5	2/96/2
KO	9/81/9	9/81/9	3/94/3
DOW	19/63/19	19/63/19	3/94/3
XOM	10/79/10	10/79/10	1/98/1
WBA	22/56/22	22/56/22	3/95/3
GS	43/13/44	34/31/35	3/95/3
HD	32/36/32	27/47/27	1/98/1
INTC	5/89/5	5/89/5	2/95/2
IBM	37/25/38	32/35/33	1/97/1
JNJ	26/48/26	21/57/21	1/99/1
JPM	9/83/9	9/83/9	0/100/0
MCD	35/31/35	30/41/29	1/98/1
MRK	11/78/11	11/78/11	2/96/2
NKE	10/79/11	10/79/11	2/96/2
PFE	5/89/5	5/89/5	2/95/2
PG	11/77/11	11/77/11	1/98/1
TRV	38/24/38	33/35/33	3/95/3
UNH	40/20/40	35/31/35	2/97/2
UTX	33/34/33	28/44/28	1/97/1
VZ	7/87/7	7/87/7	2/96/2
V	16/68/16	16/69/16	0/99/0
WMT	12/76/12	12/76/12	1/98/1
DIS	19/62/19	16/69/16	1/99/1

Table S4 Proportion of the outcome variable Y (Upwards/Stationary/Downwards) according to different α values and over August 2017.

	$\alpha = 10^{-6}$	$\alpha = 10^{-5}$	$\alpha = 10^{-4}$
AAPL	20/60/20	16/68/16	0/100/0
MSFT	6/87/7	6/87/7	0/99/0
MMM	38/24/38	29/43/29	1/98/1
AXP	15/70/15	15/70/15	1/98/1
BA	38/21/41	29/40/30	1/98/1
CAT	32/36/32	27/46/27	1/98/1
CVX	23/54/23	19/62/19	0/99/0
CSCO	5/91/5	5/91/5	2/96/2
KO	8/84/8	8/84/8	2/95/3
DOW	14/73/14	14/73/14	2/96/2
XOM	8/83/8	8/83/8	1/99/1
WBA	24/52/24	24/52/24	2/96/2
GS	43/13/43	34/32/34	2/96/2
HD	34/33/33	28/43/28	1/98/1
INTC	4/92/4	4/92/4	2/96/2
IBM	36/27/37	31/38/31	1/98/1
JNJ	30/40/30	25/49/25	1/98/1
JPM	9/81/9	9/81/9	0/99/0
MCD	34/33/34	28/43/29	1/98/1
MRK	10/80/10	10/80/10	2/97/2
NKE	10/81/10	10/81/10	2/97/2
PFE	5/90/5	5/90/5	2/95/2
PG	9/83/9	9/83/9	0/99/0
TRV	38/24/38	33/35/33	3/94/3
UNH	39/22/39	33/33/34	1/97/1
UTX	35/30/35	29/41/30	2/96/2
VZ	5/89/6	5/89/6	2/97/2
V	15/70/15	13/75/13	0/99/0
WMT	15/70/15	15/70/15	1/97/1
DIS	18/63/18	15/70/15	1/99/1

Author details
[1]Math&Stat Department at University of Victoria, Victoria, Canada. [2]Stat&Actuarial Science at University of Waterloo, Waterloo, Canada. [3]Economics Department at University of Victoria, Victoria, Canada. [4]Math&Stat Department at University of Saskatchewan, Saskatchewan, Canada.

References

1. Tay, F.E., Cao, L.: Application of support vector machines in financial time series forecasting. omega **29**(4), 309–317 (2001)
2. Huang, W., Nakamori, Y., Wang, S.-Y.: Forecasting stock market movement direction with support vector machine. Computers & operations research **32**(10), 2513–2522 (2005)
3. Chalup, S.K., Mitschele, A.: Kernel methods in finance. In: Handbook on Information Technology in Finance, pp. 655–687. Springer, Germany (2008)
4. Cortes, C., Vapnik, V.: Support-vector networks. Machine learning **20**(3), 273–297 (1995)
5. Burges, C.J.: A tutorial on support vector machines for pattern recognition. Data mining and knowledge discovery **2**(2), 121–167 (1998)
6. Hoerl, A.E., Kennard, R.W.: Ridge regression: Biased estimation for nonorthogonal problems. Technometrics **12**(1), 55–67 (1970)
7. Tibshirani, R.: Regression shrinkage and selection via the lasso. Journal of the Royal Statistical Society: Series B (Methodological) **58**(1), 267–288 (1996)
8. Zou, H., Hastie, T.: Regularization and variable selection via the elastic net. Journal of the royal statistical society: series B (statistical methodology) **67**(2), 301–320 (2005)
9. Friedman, J., Hastie, T., Tibshirani, R.: Regularization paths for generalized linear models via coordinate descent. Journal of Statistical Software **33**(1), 1–22 (2010)
